# Influence of introduced peregrine falcons on the distribution of red knots within a spring staging site

**DOI:** 10.1371/journal.pone.0244459

**Published:** 2021-01-14

**Authors:** Bryan D. Watts, Barry R. Truitt

**Affiliations:** 1 Center for Conservation Biology, William and Mary, Williamsburg, Virginia, United States of America; 2 The Nature Conservancy in Virginia, Virginia Coast Reserve, Nassawadox, Virginia, United States of America; San Diego Zoo Institute for Conservation Research, UNITED STATES

## Abstract

Predator recovery driven by single-species management approaches may lead to conservation conflicts between recovered predators and prey species of conservation concern. As part of an aggressive recovery plan, the Eastern Peregrine Falcon Recovery Team released (1975–1985) 307 captive-reared peregrine falcons (*Falco peregrinus*) and successfully established a breeding population within the mid-Atlantic Coastal Plain, a physiographic region with no historic breeding population and a critical spring staging area for migratory shorebirds. We examined the influence of resident falcons on the distribution of foraging red knots during spring migration. We conducted weekly aerial surveys (2006–2009) along the Virginia barrier islands during the spring staging period (25 April– 6 June) to map foraging red knots (*Calidris canutus*) and evaluated the influence of proximity (0–3, 3–6, >6 km) of beaches to active peregrine falcon nests on knot density (birds/km). Accumulated use of beaches throughout the season by red knots was significantly influenced by proximity of beaches to active falcon nests such that mean density was more than 6 fold higher on beaches that were >6 km compared to beaches that were only 0–3 km from active eyries. Whether or not an eyrie was used in a given year had a significant influence on the use of associated close (0–3 km) beaches. From 6.5 to 64 fold more knots used beaches when associated eyries were not active compared to when they were active depending on the specific site. Historically, red knots and other migratory shorebirds would have enjoyed a peregrine-free zone within this critical staging site. The establishment of a dense breeding population of falcons within the area represents a new hazard for the knot population.

## Introduction

Predator recovery driven by single-species management approaches often leads to unintended consequences and conservation conflicts [1–3]. Impacts may be acute when predator recovery is legally mandated and agencies have limited authority to manage downstream effects. Marshall et al. [4] outline three classes of conflicts resulting from predator recovery including “protected predator vs protected prey” where a protected predator population consumes or impacts a population of protected prey. Conflict arises when management decisions reflect single-species objectives with little consideration of the broader context of recovery.

The eastern population of peregrine falcons (*Falco peregrinus*) experienced a precipitous decline throughout the 1950s [5], was believed to have been extirpated by the early 1960s [6] and was listed as federally “endangered” in June 1970 (50 CFR 17.11–17.12). In 1975, the U.S. Fish and Wildlife Service appointed an Eastern Peregrine Falcon Recovery Team to develop and implement a Recovery Plan [7]. As part of this plan, the team released 307 captive-reared falcons (1975–1985) on artificial structures within the mid-Atlantic Coastal Plain [7,8], a physiographic region with no historic breeding population. The decision was based on the fact that both prey availability and fledgling return rates were higher on the Coastal Plain compared to the historic mountain range [9]. The effort followed a single-species set of objectives with no consideration of the broader community. By 2007 the population had reached 55 breeding pairs (all nesting on artificial substrates) and was self-sustaining [10]. Pairs on the outer coast have adjusted their breeding phenology to match the period of highest metabolic demand to the peak passage of shorebirds. Diet during the brood-rearing period is dominated by migrating shorebirds including red knots [11,12].

The *rufa* subspecies of the red knot (*Calidris canutus*) has declined from an estimated population size of 100,000–150,000 individuals to possibly fewer than 30 000 over a period of just thirty years [13,14]. Concern for the population has led the United States Fish and Wildlife Service to list the form as federally “threatened” (16 U.S.C. 1531 et seq.). The action follows its listing as an endangered population in Canada [15] and its declaration of endangerment by the Convention on the Conservation of Migratory Species of Animals [16]. The *rufa* population undertakes dramatic migratory movements throughout the Western Hemisphere from high-Arctic breeding grounds to winter areas on the southern tip of South America [13,17]. The population uses a network of staging areas to refuel along migration routes [18]. One strategically critical site is the mid-Atlantic Coast from New Jersey south to the mouth of the Chesapeake Bay where many birds refuel for the last time before making nonstop flights to Arctic breeding grounds [19,20]. The Virginia barrier islands support several thousand red knots during spring migration [20]. During some years, these birds represent a considerable (>25%) portion of the overall population known to stage along the Atlantic Coast [13]. Staging within this area is compressed within a three-week window [21,22] and birds must achieve a sufficient leaving weight (often 50% above arrival mass) in order to have enough energy reserve to complete their final flight to the Arctic and arrive with enough surplus to initiate reproduction [23,24]. In order to reach energetic objectives, birds must maintain very high intake rates [13,25].

Peregrine falcons and migratory shorebirds have a nearly global association that has shaped migratory strategies and distribution of both predator and prey populations [26,27]. Although peregrines may have a direct impact on some populations via increased mortality rates [28–30], the much greater impacts are manifest via non-lethal adjustments in behavior. Peregrines have been suggested to shape shorebird migration routes and winter distributions [26,31,32] and their presence has been implicated in observed changes in the length of stay and refueling schedules within staging sites [27], shifts in foraging sites [33] and schedules [34], altered patterns in the use of communal roosts [35,36] and reductions in peak body weights/wing loading [37]. The influence of peregrines on shorebird distribution and behavior has become increasingly apparent as peregrine populations throughout the globe have recovered from contaminant-induced lows [27,32].

The decision to establish breeding falcons within a critical mid-Atlantic staging area may have changed conditions for red knots. Historically, staging red knots would have enjoyed a peregrine-free zone during this critical staging window since the physiographic region supports no natural cliffs for nesting peregrines and over wintering or migrant falcons would have already left for northern breeding grounds. Resident falcons may influence a wide range of behaviors (e.g. time budgets, foraging schedules, flocking behavior) that are relevant to a successful staging cycle for red knots. Here, we test whether or not resident falcons are having an influence on space use by staging red knots.

## Methods

### Study area

**Fig 1:**
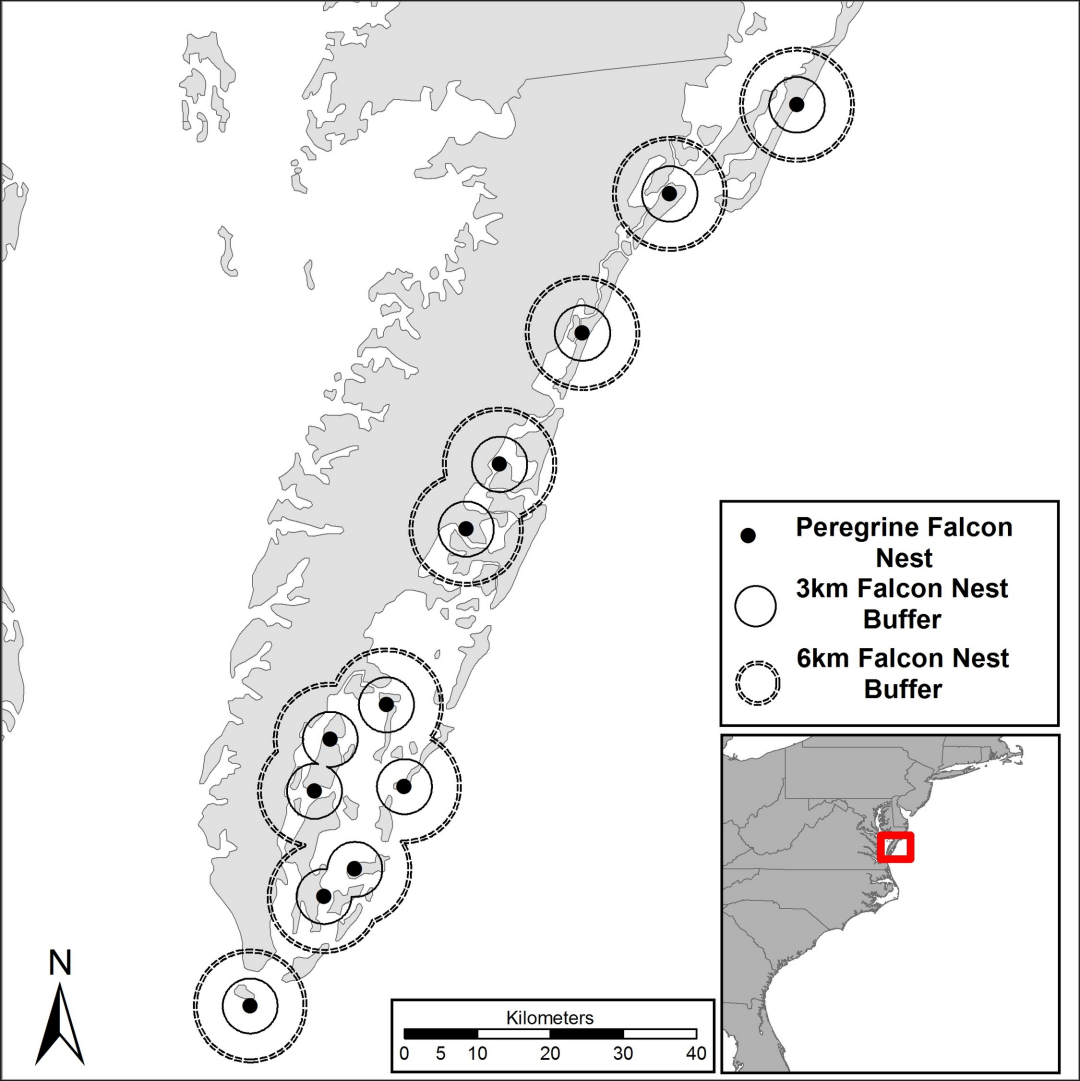
Map of study area with peregrine falcon eyries and buffer boundaries used to delineate beaches within different proximity categories. Red knot surveys were flown along barrier island beaches that lie along the Atlantic Ocean. Base map layer is credited to the National Atlas of the United States of America.

The Virginia barrier islands (Fig 1) are located along the seaward margin of the Delmarva Peninsula (centered on 37° 56’ N, 75° 61’ W) and represent the most pristine chain of barrier islands remaining along the Atlantic coast. The chain contains 14 primary islands and numerous bars, spits, and shoals from Assateague National Seashore south to Fisherman Island National Wildlife Refuge. These islands are subjected to an average of 38 extratropical storms annually with enough intensity to rework beach sand and, as a result, have the highest beach erosion rate of any location along the Atlantic coast [38,39]. Most of the islands are remote and accessible only by boat, providing some protection from human disturbance. A large portion (>95%) of this system is in protective ownership by The Nature Conservancy, The U.S. Fish and Wildlife Service and the Commonwealth of Virginia. The study area is a terminal spring staging site for several shorebird species that build fat reserves before moving on to arctic breeding grounds [40,41]. Red knots use the active beach zone along the islands for foraging [20]. The study area is also a restoration site for peregrine falcons and annually supports 8–12 breeding pairs that nest on artificial structures [42].

### Knot surveys

We determined the distribution of staging red knots along the Virginia barrier islands using a series of low-altitude aerial surveys from the last week of April through the first week of June 2006–2009. Each survey began on Assateague Island (Virginia/Maryland border) and followed the outer beaches south to Fisherman Island. The surveys included all active beach zones of the outer barrier system (approx. 141 km of open beach). We flew 6 survey flights each year beginning the last week of April and ending the first week of June. We conducted flights on a falling tide initiated around half stage. The decision to conduct surveys around low tide reflects our interest in the impact of peregrines on the distribution of foraging rather than roosting knots.

We conducted all surveys from a Cessna 172 (Cessna, Wichita, KS), high-wing aircraft flying 25–30 m above the ground at an air speed of approximately 140 km/hr. We used low altitude flights to temporarily flush birds to ease identification and numerical estimation. We believe that disturbance was minimal as flocks flushed circled and resettled in seconds. We flew a line on the outer edge of the surf zone to encourage birds to flush inland. Surveys were a collaborative effort between 2 observers (the same 2 observers conducted all surveys). The first observer identified bird species and estimated flock sizes while the second observer mapped flocks on aerial photographs. We mapped each flock and gave each a unique code to cross-reference with survey data. We recorded all survey data in digital audio files and later transcribed them to data sheets. Work with red knots was observational only and no Institutional Animal Care permit was required.

### Peregrine monitoring

We have monitored the nesting population of peregrine falcons annually throughout the study area since the establishment of the first breeding pair in 1979 [42]. We monitor sites determined to be occupied during a given year 2–5 times from March through July to document breeding activity. We consider a breeding territory to be occupied if a pair of adult peregrines is resident during the breeding season and to be active if eggs or young are observed within the nest site. Peregrine falcons were captured and handled under the Institutional Animal Care and Use Committee protocol IACUC-2017-04-18-12065 of The College of William and Mary and were banded under permit #21567 issued by the United States Geological Survey.

#### Data analysis

We evaluated the influence of active peregrine falcon nesting towers on red knot distribution by quantifying the density (birds/km of shoreline) of foraging knots within three proximity categories (0–3, 3–6, >6 km). Proximity categories were loosely based on information on falcon home range during the breeding season [43] but were also tailored to represent a distance gradient across the study landscape. We initially conducted a sensitivity analysis across a range of potential distance categories and determined that this structure provided the most even distribution of beach length within categories. We extended concentric rings from all active towers and overlaid boundaries on beach habitat throughout the study area in order to classify beach segments according to proximity categories (Fig 1).

Aerial survey maps were then used to associate red knots with shoreline segments. The number of knots detected were summed according to proximity categories and divided by the total shoreline length within categories to produce knot densities (birds/km) for each weekly survey (N = 6) for all years (2006–2009). We digitized island and segment lengths from high-resolution, true-color aerial photographs taken during low tide for each year using ArcView 3.2 (Environmental Systems Research Institute, Inc., Redlands, CA).

We evaluated temporal (within season) and spatial (proximity to peregrine eyries) patterns in the density of red knots within the study area using a 2-way analysis of variance [ANOVA] where season (six ranks; weekly surveys) and proximity (three ranks; 0–3, 3–6, >6 km) were grouping parameters and surveys were samples. We compiled the number of red knots along beaches for a given day to represent the dependent parameter. We also evaluated the accumulated use of beaches within proximity categories using a 1-way ANOVA with each year as a sample. We defined accumulated use as the sum of 6 surveys (24 April-6 June) for each year. We found no effect of year on either seasonal or accumulated densities and dropped this parameter on subsequent analyses.

We examined the influence of eyrie activity status on the use of close beaches (0–3 km). We define active eyries as having a pair of resident falcons and observations of eggs or young in the nest. Five of the 12 peregrine eyries were not active for at least one year during the study period. We compared the frequency (sum for the survey year) of red knots observed on close beaches between years that were classified as active and not active using frequency statistics (G-test) with an equal distribution as the null model.

## Results

**Fig 2:**
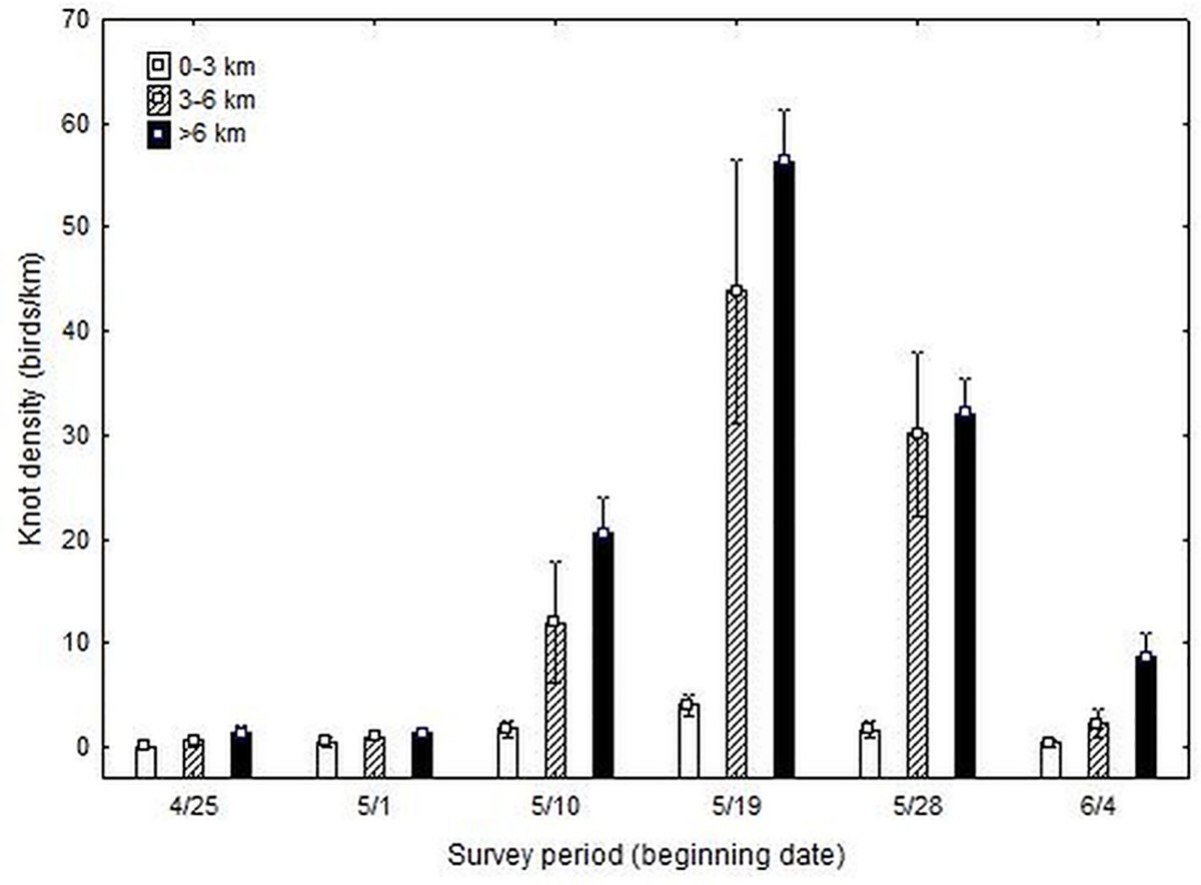
Results of weekly aerial surveys (2006–2009) for red knots along the Virginia barrier islands. Knots were mapped and assigned to beach segments according to their proximity (0–3, 3–6, >6 km) to active peregrine falcon eyries. Knot densities were calculated for proximity categories for each survey (6 weekly surveys/year). Means and standard errors are presented for weeks and proximity categories across years.

We mapped more than 60 000 red knots along the Virginia barrier islands during 24 aerial surveys. Knots began arriving in early May, reached peak numbers during the third week of May and declined through the first week of June (Fig 2).

Week of survey and proximity to an active falcon nest had significant influences on knot density and there was a significant week by proximity interaction (Table 1). The significant interaction term appears to be driven by the seasonal pattern in the close (0–3 km) category. Although the medium (3–6 km) and the far (>6 km) categories have very similar phenology patterns, use of the close category is lower early and higher later than expected.

**Table 1:** Results of a 2-way analysis of variance (ANOVA) showing effects of survey week and proximity (0–3, 3–6, >6 km) to active falcon nest on red knot density (birds/km) along the Virginia barrier islands in (2006–2009).

Source	SS	df	MS	*F*	*P*
Week	11,109	5	2,222	23.3	<0.001
Proximity	4,609	2	2,304	24.2	<0.001
Week x Proximity	4,728	10	473	5.0	<0.001
Error	5,715	60	95		

SS represents sum of squares and MS represents mean square.

**Fig 3:**
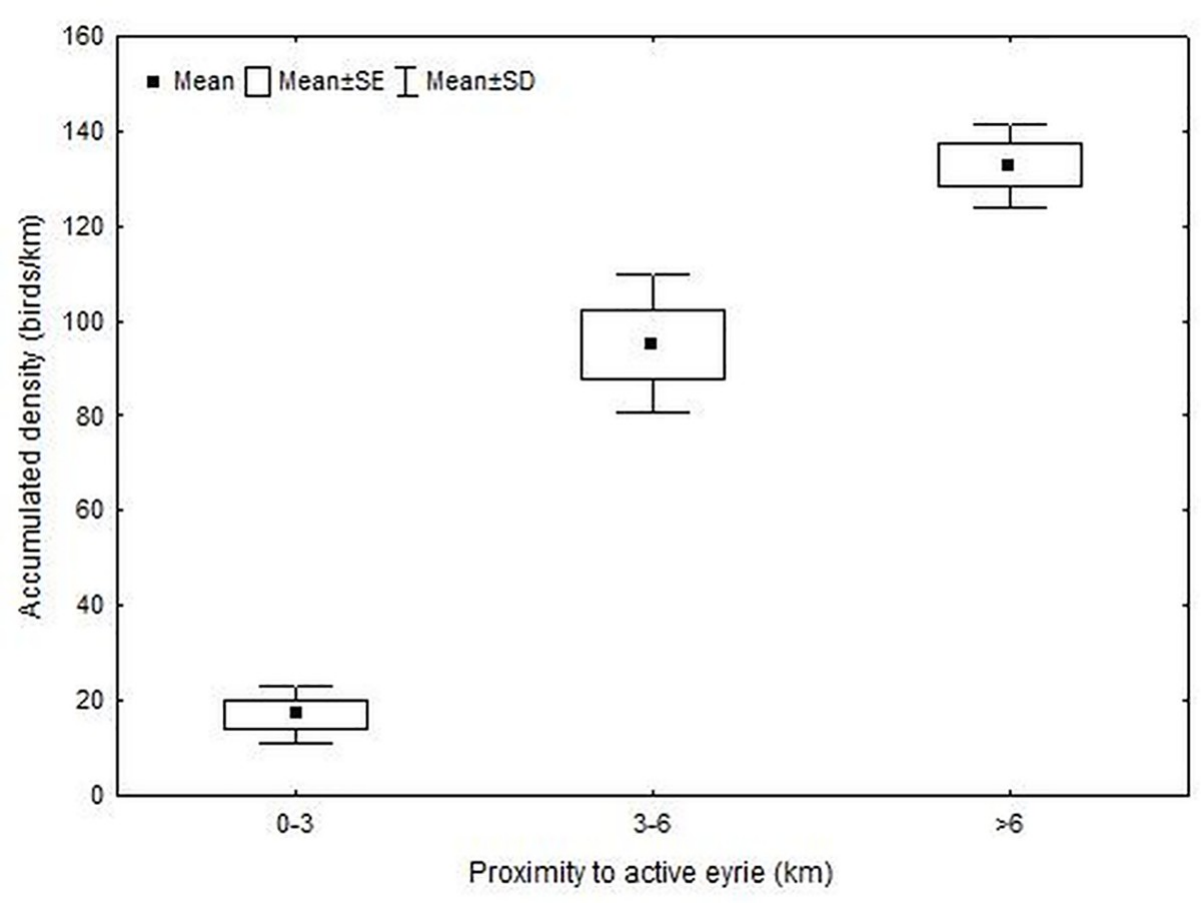
Accumulated density of red knots along beaches according to their proximity (0–3, 3–6, >6 km) to active peregrine falcon eyries. Accumulated densities represent the sum of all surveys (6 weekly surveys/year) within each year divided by the length of beaches within proximity categories. Means and standard errors are presented for proximity categories across years.

Accumulated use of beaches by red knots was significantly influenced by proximity of beaches to active falcon nests (1-way ANOVA: *F*_2,9_ = 127.5, *P* < 0.001; (Fig 3). Accumulated density was more than 6 fold higher on beaches that were >6 km compared to beaches that were only 0–3 km from active eyries. Beaches that were intermediate (3–6 km) distances from active eyries supported 5 fold higher densities compared to close beaches.

The activity status of peregrine falcon eyries had an influence on the use of beaches in the close (0–3) category. Of the 12 eyries used by peregrines during the study period 5 were not active every year (Table 2). Although we found considerable variation in knot use between beaches associated with different eyries, frequency of knot use was significantly influenced by activity status for all sites (Table 2). From 6.5 to 64 fold more knots used beaches when associated eyries were not active compared to when they were active depending on the specific site.

**Table 2:** Results of surveys for red knots around peregrine falcon territories that varied in activity status through the study period (2006–2009).

Territory	Year	Status	N	Density (N/km)	G-statistic	P
Assateague	2006	Not Active	259	43.6		
Assateague	2007	Not Active	394	66.3		
Assateague	2008	Active	49	8.2		
Assateague	2009	Not Active	305	51.3	324.3	<0.001
Wallops	2006	Active	191	31.0		
Wallops	2007	Active	236	38.3		
Wallops	2008	Not Active	2161	350.8		
Wallops	2009	Not Active	1080	175.3	2,818.4	<0.001
Metompkin	2006	Active	15	2.5		
Metompkin	2007	Active	30	4.9		
Metompkin	2008	Active	10	1.6		
Metompkin	2009	Not Active	1175	193.3	2,851.5	<0.001
Godwin	2006	Active	35	18.0		
Godwin	2007	Active	45	23.2		
Godwin	2008	Not Active	395	203.6		
Godwin	2009	Active	72	37.1	550.2	<0.001
Fisherman	2006	Active	63	6.8		
Fisherman	2007	Not Active	196	21.1		
Fisherman	2008	Not Active	870	93.4		
Fisherman	2009	Not Active	350	37.6	978.6	<0.001

N refers to the number of red knots counted along beaches that were 0–3 km from falcon eyries. Status refers to whether or not the breeding territory was occupied during a given year.

## Discussion

The establishment of a dense breeding population of peregrine falcons within the most significant terminal spring staging area represents a new hazard for the *rufa* population. Proximity of beaches to active falcon nests had a significant influence on their use by red knots throughout the season. Within the study area, observations of peregrines are common along foraging beaches, flocks of knots are regularly flushed by peregrines while foraging and shorebirds including knots are the dominant prey recovered from nesting boxes [12]. Sensitivity of knots to the presence of falcons was demonstrated by the significant difference in the use of beaches according to whether or not specific eyries were active during a given year. These results are consistent with previous studies that have implicated the presence of falcons in shifting shorebird behavior or habitat use within other migratory staging areas [33,44,45] or on the winter grounds (e.g. [34,46,47]).

Red knots use patches of exposed peat and open sand beaches as foraging habitat along the barrier islands [20]. The dominant prey used by knots within these two habitats include blue mussels (*Mytilus edulis*) for peat patches and coquina clams (Donax variabilis) for sandy beaches [48]. Intertidal bivalves are recruitment driven where larval settlement patterns are notoriously influenced by fluctuations in the physical environment [49–51], leading to dramatic spatio-temporal variation in standing stocks (e.g., [52,53]). Red knots exhibit wide year-to-year variation in the relative use of the two habitats [20], presumably reflecting the underlying population dynamics of the two bivalve populations. Annual variation in bivalve stocks may help to explain differences in the magnitude of site-to-site responses to occupancy of specific nesting towers.

One of five major factors emphasized as causing red knot declines in the final listing rule (79 FR 73706) was habitat loss [54]. The decision to establish a peregrine population outside of the historic breeding range as a single-species management tool is having an impact on the capacity of the area to service staging red knots. Reductions in knot densities on close and intermediate beaches compared to distant beaches suggest that falcons may restrict access to foraging habitats around active eyries. This impact is similar to the effect of coastal development [55] or direct human disturbance [56] on the number of staging knots that may be supported by available foraging habitat. The actual impact of falcons on the number of knots supported throughout the study area or their ability to reach leaving weights is unclear from the existing data but may be worth further investigation. Of particular interest is the time budget of nesting peregrines along barrier beaches and how beach use may impact the foraging rates of knots.

For some migratory shorebirds, tradeoffs between predation-danger and foraging rates have been suggested to extend beyond localized adjustments in behavior or habitat choice to larger scale changes in migration routes or shifts in the use of staging sites [26,57]. Establishment of nesting peregrine falcons within the mid-Atlantic Coastal Plain has occurred over the past three to four decades with the population reaching 55 pairs by 2007 [20] and around 85 pairs by 2018 [58,59]. Peregrine falcons breeding on the outer coast are currently confined to areas from Virginia north along the Atlantic Coast. South of Virginia, the outer coast continues to represent a peregrine-free zone during the critical window for spring staging. With the increase in predation danger along the mid-Atlantic coast it is possible that an increased number of knots are stopping along the coast to the south for their final staging event. Such a shift would increase the flight distance between final staging area and the breeding grounds and may also include challenges of refueling under poorer foraging conditions. Although significant numbers of knots have been staging within this area in recent years [60], we have no historic survey data on a large enough scale to assess whether or not declines along the mid-Atlantic Coast have been offset by numbers along the South Atlantic Coast. We do know that declines documented in Delaware Bay are consistent with declines observed within the largest overwintering site, Tierra del Fuego [61]. Large-scale survey efforts are needed that may address regional shifts in the use of staging sites.

Ultimately, the management of peregrine falcons along the mid-Atlantic coast should transition from single-species to ecosystem-based management [62,63] where staging shorebirds and other species are considered. In recent years, the conservation community has attempted to mitigate the impact of peregrine falcons on staging knots within the study area with mixed results. Three of the towers closest to beaches were taken down in an attempt to dissolve nesting territories. One of the pairs moved to a bridge within the territory and continues to nest, one of the pairs nested on the ground in the dunes for three years [64] and one of the pairs disappeared. Pairs appear to have high site fidelity and other structures (e.g., abandoned shacks, chimneys, duck blinds) provide alternative nest sites. However, removal of towers may disperse peregrine pairs over time and reduce pressure on shorebirds staging on outer beaches. A second strategy has been to reduce the metabolic demand of falcon broods within the study area by translocating young to the mountains [59]. Since 2000, more than 300 young falcons have been moved from the Coastal Plain to be hacked within the mountains. Falcons are suitable for hacking when they reach 25–30 days of age. Removing falcon broods from the area reduces their metabolic demand on the system by approximately 50% [12]. Execution of this strategy serves to 1) reduce pressure on staging shorebirds while 2) restoring peregrines to their historic mountain range. Expanding the translocation program would further reduce metabolic demand and relieve pressure on red knots and other migratory shorebirds.
